# Deficiency of protein-L-isoaspartate (D-aspartate) *O*-methyl-transferase expression under endoplasmic reticulum stress promotes epithelial mesenchymal transition in lung adenocarcinoma

**DOI:** 10.18632/oncotarget.24324

**Published:** 2018-01-27

**Authors:** Masahiro Yamashita, Masahito Ogasawara, Yasushi Kawasaki, Miyuki Niisato, Heisuke Saito, Shuya Kasai, Chihaya Maesawa, Makoto Maemondo, Kohei Yamauchi

**Affiliations:** ^1^ Department of Pulmonary Medicine, Allergy and Immunological Diseases, School of Medicine, Iwate Medical University, Morioka, Iwate, Japan; ^2^ Department of Pharmacology, Graduate School of Medicine, Ehime University, Toon, Ehime, Japan; ^3^ Department of Health Chemistry, School of Pharmacology, Iwate Medical University, Shiwa, Iwate, Japan; ^4^ Department of Cancer Biology, Iwate Medical University, Shiwa, Iwate, Japan; ^5^ Department of Biomolecular Sciences, Graduate School of Life Sciences, Tohoku University, Sendai, Miyagi, Japan; ^6^ Geriatric Health Services Facilities, Keiyu, Morioka, Japan

**Keywords:** lung adenocarcinoma, PIMT, endoplasmic reticulum stress, epithelial mesenchymal transition, hypoxia-inducible factor 1α

## Abstract

A prognostic association between the novel chaperone protein-L-isoaspartate (D-aspartate) *O*-methyltransferase (PIMT) and lung adenocarcinoma has recently been reported. Here, we evaluated the functional roles of PIMT in the progression of lung adenocarcinoma. PIMT expression was detectable in 6 lung adenocarcinoma cell lines: A549, H441, H460, H1650, Calu 1, and Calu 6 cell lines. In A549 and H441 cells, knockdown by PIMT using silencing RNA of PIMT (si-PIMT) and/or small hairpin-RNA (sh-PIMT) induced a decrease in the expression of E-cadherin with an increase in vimentin expression, indicating that the epithelial to mesenchymal transition (EMT) was induced. Cell mobility, including migration and invasion capability, was increased in sh-PIMT A549 stable and si-PIMT H441 cells compared to in control cells. Endoplasmic reticulum (ER) stress, such as Thapsigargin (Tg) stress and hypoxia, induced EMT in A549 cells but not in other cell types, with an increase in GRP78 expression, whereas overexpression of PIMT reduced the EMT and cell invasion under stress conditions. The expression of hypoxia inducible factor-1 alpha (HIF1α) and Twist increased in sh-PIMT A549 and si-PIMT H441 cells, and Tg stress increased HIF1α expression levels in A549 cells in a dose-dependent manner. Moreover, LW6, an HIF1α inhibitor, reduced EMT, cancer invasion, and the levels of Twist in sh-PIMT A549 cells. Our results indicate that deficiency of supplemental PIMT expression under ER stress facilitates EMT and cell invasion in some cell types of lung adenocarcinoma.

## INTRODUCTION

Lung cancer is a major global health concern, and non–small cell lung cancer is the most common disease type [1]. Despite advances in chemotherapy, radiation, and surgery, the prognosis of lung cancer remains unsatisfactory [2]. Carcinogenesis, tumorigenesis, invasion, and distant metastasis of cancer cells are involved in cancer development [3, 4]. Various stresses, such as oxidant stress, malnutrition, and hypoxia within tissue, are exerted on cancer cells through these processes and can cause the accumulation of unfolded proteins in the ER [5–7]. Molecular chaperones can fold various types of unfolded proteins induced by ER stress. A large number of studies has shown that molecular chaperones show increased expressed in cancer cells, and it has been speculated that molecular chaperones facilitate the survival of cancer cells via anti-apoptotic effects [8, 9].

Recently, concern has increased regarding the pathogenic association between cancer and protein L-isoaspartyl (D-aspartyl) *O-*methyltransferase (PIMT), which functions as a chaperone for the conversion of isomerized L-isoaspartyl and D-aspartic acid residues into L–Asp [10–13]. Lapointe *et al.* found that expression levels of PIMT are inversely correlated with stage progression in astrocytic tumors [10]. In contrast, Lee *et al.* showed that higher PIMT expression is associated with poor prognoses for breast and lung cancers [11]. They demonstrated mutual interference between PIMT and wild-type p53 expression *in vitro* using various cell lines, and found that inductive expression of PIMT exerts an anti-apoptotic and carcinogenic effect by inhibiting the wild-type p53 pathway. We previously reported that strong expression of PIMT was immunohistochemically detected in approximately half of patients with lung adenocarcinoma and an independent predictor of poor prognosis for lung adenocarcinoma [13]. In addition, strong PIMT expression was correlated with higher levels of 78-kDa glucose-regulated protein (GRP78), a marker of ER stress, rather than p53 expression. However, it has remains unclear whether the inconsistent prognostic values of higher PIMT expression are related to specific types of cancers and the roles of PIMT in multiple processes during the development of each type of cancer.

In the present study, we evaluated the functional roles of PIMT in the disease progression of lung adenocarcinoma using several cell lines, based on the hypothesis that PIMT expression participates in cancer progression of lung adenocarcinoma rather than carcinogenesis. We found that inhibition of PIMT expression using small interference (si)-RNA and small hairpin (sh)-RNA resulted in epithelial mesenchymal tradition (EMT) in some of the cell lines. Our results provide insight into the pathogenesis of lung adenocarcinoma.

## RESULTS

### PIMT expression in cancer cell lines and epithelial properties in si-PIMT cancer cells

**Figure 1:**
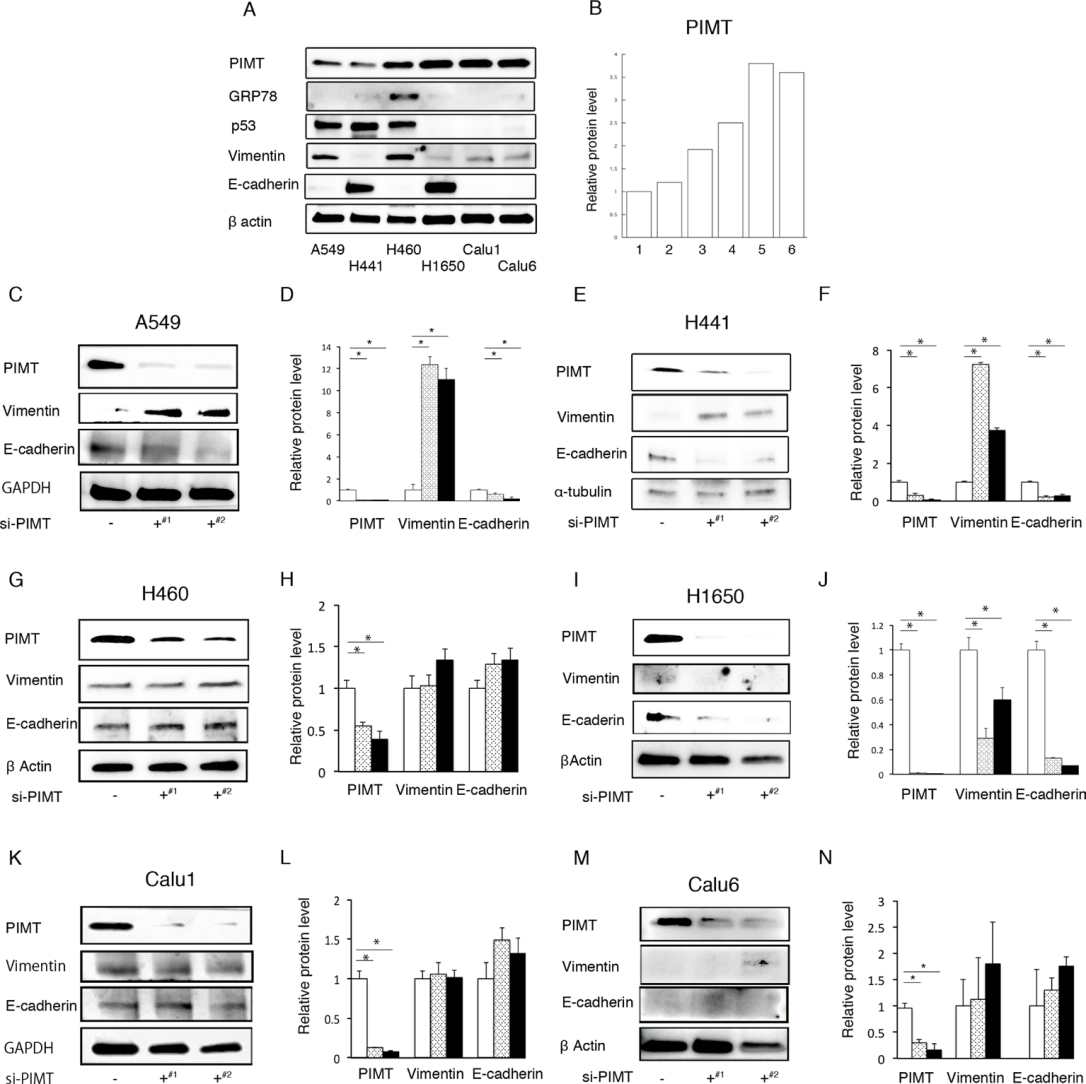
PIMT expression in cancer cell lines and epithelial properties in si-PIMT cancer cells (**A**) Immunoblotting of PIMT, GRP78, p53, vimentin, and E-cadherin in 6 lung adenocarcinoma cell lines: A549, H441, H460, H1650, Calu 1, and Calu 6. (**B**) Expression levels of PIMT in the six cell lines. (**C, D**) Immunoblot and intensity levels of PIMT, vimentin, and E-cadherin in A549 cells interfered by PIMT si-RNA anti-sense (J-010000-05-0002^#1^ and J-010000-07-0002^#2^). Immunoblot and intensity levels of vimentin, E-cadherin, and PIMT in H441 (**E, F)**, H1650 (**G, H**), H460 (**I, J**), Calu1 (**K, L)** and Calu6 cells (**M, N**) interfered by PIMT si-RNA anti-sense (J-010000-05-0002^¶^ and J-010000-07-0002^§^). ^*^indicates *p* < 0.05.

We explored the expression of PIMT in 6 lung adenocarcinoma cells lines: A549, H441, H460, H1650, Calu 1, and Calu 6 cells (Figure 1A and 1B). A549 and H441 cells showed lower levels of PIMT expression than the other 4 cell lines. GRP78 expression was detected in H460 cells, but weakly expressed in the remaining lineages. p53 expression was remarkably decreased in H1650, Calu 1, and Calu 6 cells, while expression was detected in A549, H441, and H460 cells. Vimentin expression was increased in A549 and H460 cells compared to in other cells, while H441 and H1650 cells showed higher levels of E-cadherin expression. Two anti-sense PIMT si-RNAs (J-010000-05-0002 and J-010000-07-0002) induced a significant decrease in E-cadherin expression and increase in the expression of vimentin in A549 and H441 cells, indicating that EMT occurred (Figure 1C–1F). H1650 cells showed a significant decrease in E-cadherin and vimentin expression (Figure 1I and 1J). No change in vimentin and E-cadherin expression was observed in the remaining 3 cell lines, which showed a higher intensity of PIMT expression (Figure 1G, 1H, and 1K–1N). Si-PIMT H441 cells morphologically showed minimal changes, when compared with si-control cells, although si-PIMT A549 cells showed weaker connection with neighboring cells relative to si-control A549 ones (Supplementary Figure 1).

### Mobility capability in si-RNA PIMT A549, H441, and H1650 cells

**Figure 2:**
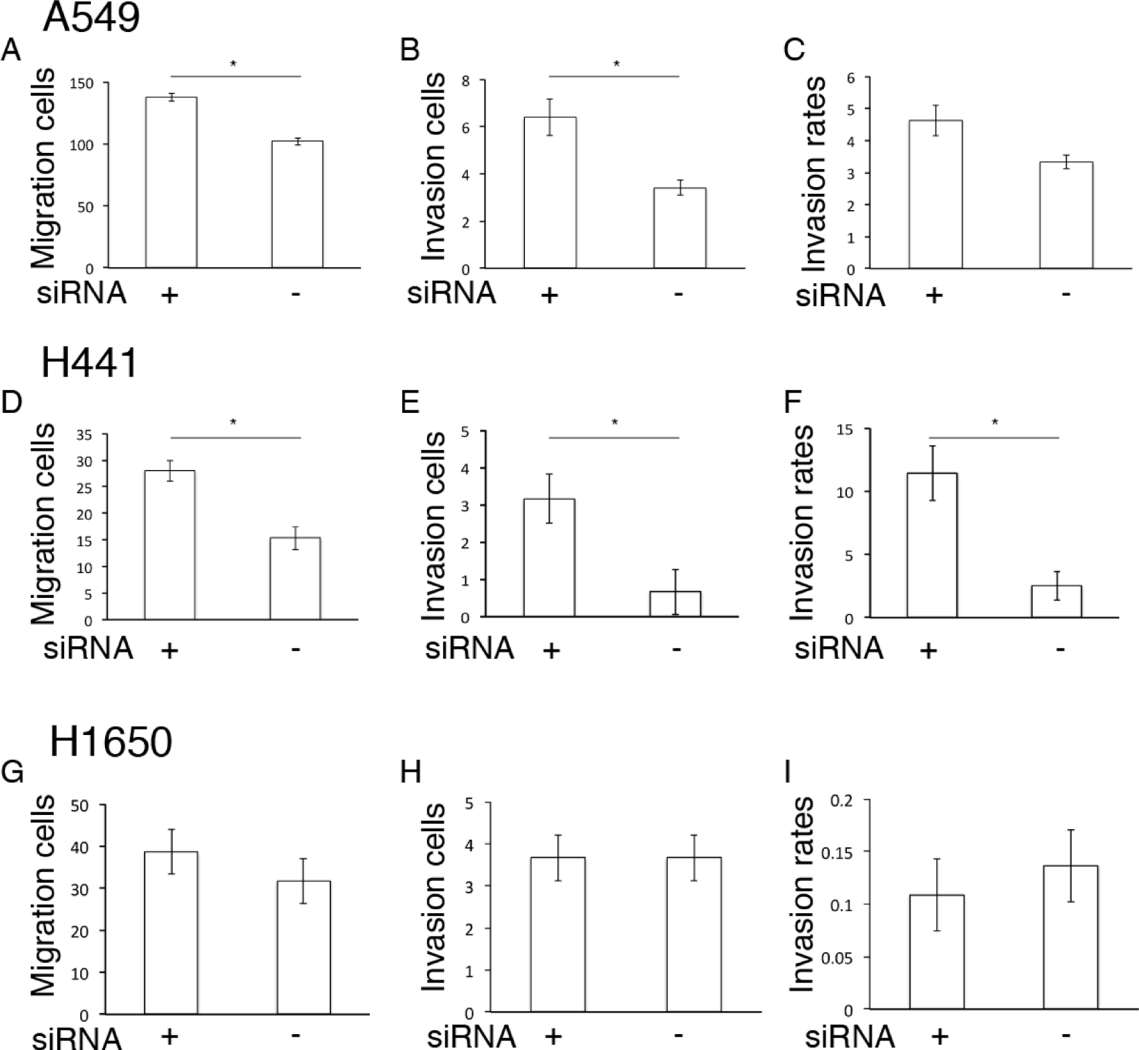
Mobility capability in si-RNA PIMT A549, H441 and H1650 cells Comparison of migration and invasion capabilities between si-PIMT and si-control A549 cells (**A–C**), H441 (**D–F**) and H1650 cells (**G–I**). ^*^indicates *p* < 0.05.

Next, we estimated mobility capability in si-PIMT A549, H441 and H1650 cells in a Matrigel gel assay. Si-PIMT A549 and H441 cells showed increased migration and invasion capabilities relative to si-control cells, although si-PIMT H1650 showed no significant difference (Figure 2). These results indicated that PIMT expression is correlated to the conservation of epithelial properties and mobility in A549 and H441 cells.

### Epithelial and mobility properties on sh-RNA PIMT A549 lines

**Figure 3:**
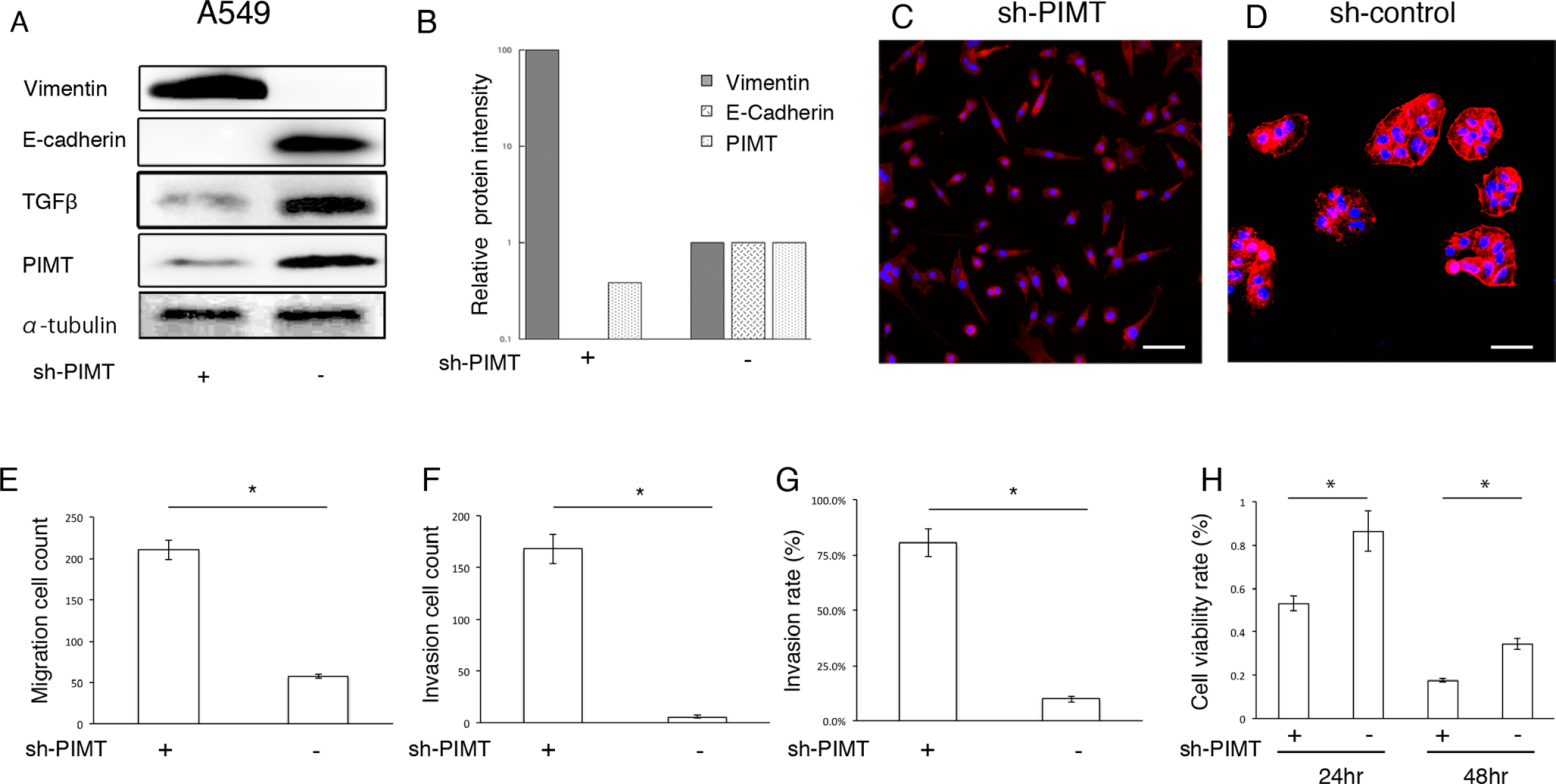
Epithelial properties and mobility capability in sh-PIMT A549 cells (**A, B**) Immunoblot and intensity levels of PIMT, vimentin, E-cadherin, and TGFβ in sh-PIMT and sh-control A549 cells. (**C, D**) Morphologic differences between sh-PIMT and sh-control A549 cells. Scale bar, 60 μm. (**E–G**) Differences in migration and invasion capabilities between sh-PIMT and sh-control A549 cells. (**H**) Differences in proliferation rate between A549 sh-PIMT and sh-control cells. A549 cells were treated with 8.0 µg/mL of cisplatin. ^*^ indicates *p* < 0.05.

Further, we constructed sh-PIMT and sh-control cells in the A549 cell line. Consistently, sh-PIMT A549 cells showed a clearer decrease in E-cadherin expression and increase in the expression of vimentin compared to control cells (Figure 3A and 3B). Sh-PIMT A549 cells showed spindle-like shapes compared with the sh-control (Figure 3C and 3D). Migratory and invasive capabilities were significantly increased in sh-PIMT A549 cells compared to in sh-control cells (Figure 3E–3G). In contrast, sh-PIMT A549 cells showed a significant decrease in cell proliferation following treatment with 8.0 µg/mL cisplatin compared to sh-control cells (Figure 3H). Although TGFβ has been reported to induce EMT in A549 cells, the expression of TGFβ was increased in A549 sh-control cells compared to in A549 sh-PIMT cells, indicating that PIMT knockdown-induced EMT in A549 occurred independently of TGFβ [14].

### Response of lung adenocarcinoma cell lines to Thapsigargin and tunicamycin

**Figure 4:**
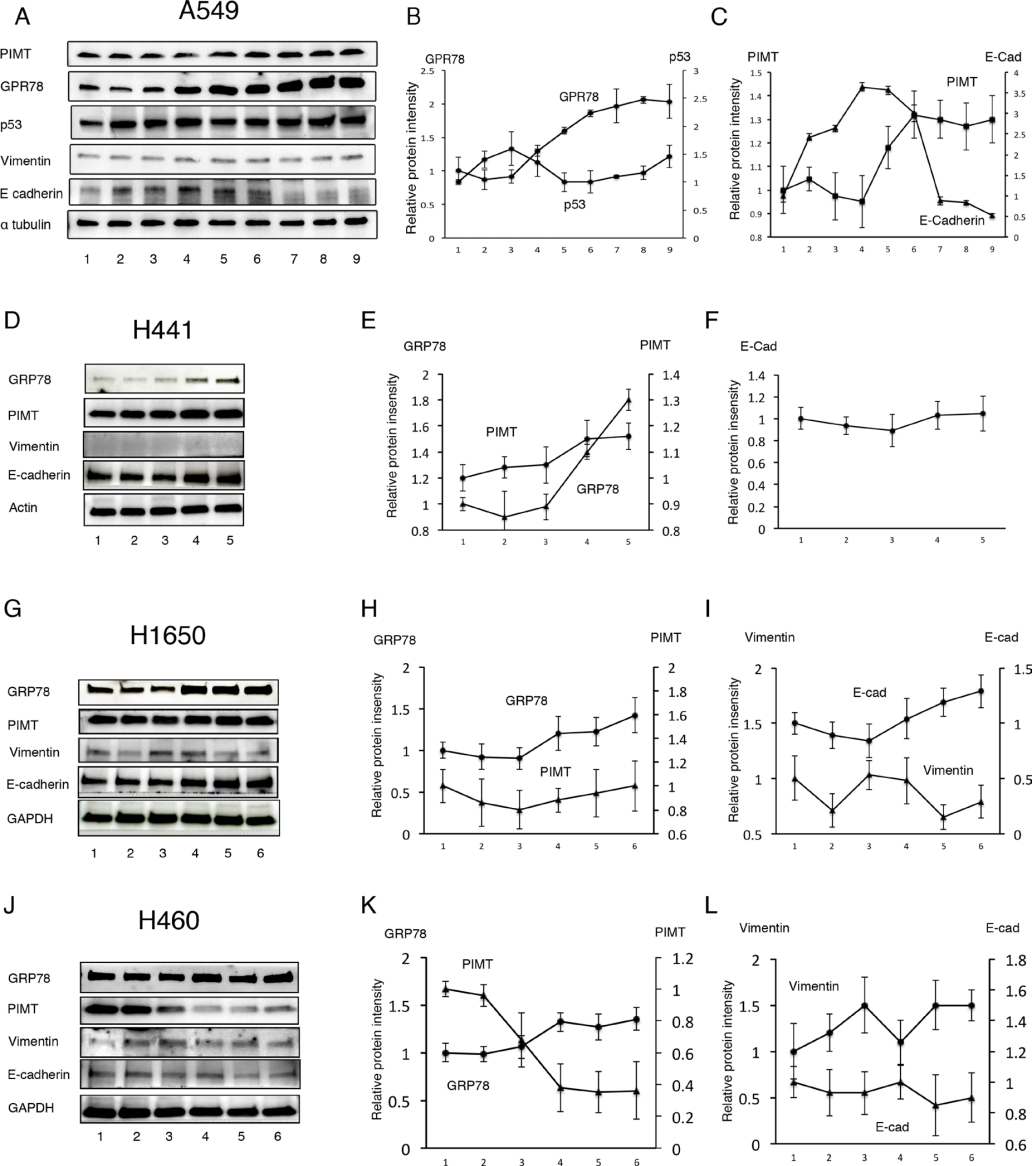
Response of lung adenocarcinoma cell lines to Thapsigargin (**A**) Immunoblotting of GRP78, PIMT, p53, vimentin, and E-cadherin in A549 cells treated with Thapsigargin (Tg). Line 1, DMSO; Line 2, 1.0 × 10^–4^ µM of Tg; Line 3, 1.0 × 10^–3^ µM of Tg; Line 4, 5.0 × 10^–3^ µM of Tg; Line 5, 1.0 × 10^–2^ µM of Tg; Line 6, 5.0 × 10^–2^ µM of Tg; Line 7, 0.1 µM of Tg; Line 8, 0.2 µM of Tg; Line 9, 0.5 µM of Tg. (**B**) Intensity of GRP78 and p53 in A549 cells treated with Tg. (**C**) Intensity of PIMT and E-cadherin in A549 cells treated with Tg. Immunoblotting and relative intensity of GRP78, PIMT, vimentin, and E-cadherin in H441 (**D–F**), H1650 (**G–I**), and H1650 (**J–L**) cells treated with Tg. Line 1, DMSO; Line 2, 1.0 × 10^–4^ µM of Tg; Line 3, 1.0 × 10^–3^ µM of Tg; Line 4, 1.0 × 10^–2^ µM of Tg; Line 5, 0.1 µM of Tg; Line 6, 1.0 µM of Tg.

Next, we explored whether the conservative epithelial effects by PIMT expression are attributable to standard chaperone functions using two types of ER stress inducers, Tg and Tn. Over a range of Tg concentrations from 1.0 × 10^–4^ to 0.5 µM, GRP78 protein levels in A549, H441, and H1650 cells were increased in a dose-dependent manner (Figure 4A–4I). In contrast, H460 cells showed no change in GRP78 in response to Tg (Figure 4J and 4L). PIMT expression was slightly increased in A549 and H441 cells at Tg concentrations above 1.0 × 10^–2^ µM relative to baseline levels, although no change was observed in H1650 cells. PIMT expression in H460 cells was decreased at Tg concentrations above 1.0 × 10^–2^ µM. Vimentin expression showed minimal changes in all cell lines. E-cadherin levels in A549 cells were significantly decreased compared to baseline at Tg concentrations above 0.1 µM, although the levels were increased compared to those at baseline at a concentration of 5.0 × 10^-2^ µM of Tg. In contrast, E-cadherin levels showed no change in H441 and H460 cells in response to Tg stress, and was somewhat increased in H1640 cells. Tg stress had a limited influence on p53 expression (Figure 4A and 4B). In addition, Tn did not decrease E-cadherin expression in A549 and H441 cells, although Tn induced an increase in GRP78 expression in these cells in a dose-dependent manner (Supplementary Figure 2). Taken together, partial EMT was detectable only for the combination of A549 cells and Tg.

### Overexpression of PIMT protect Tg-induced EMT in A549 cells

**Figure 5:**
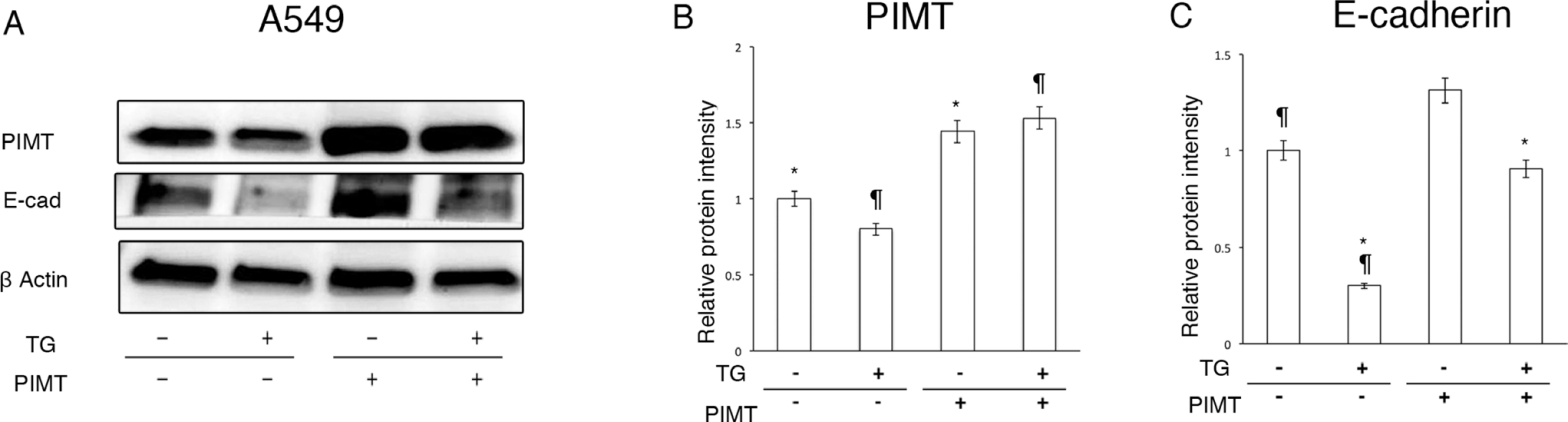
Supplemental expression of PIMT reduces EMT and cell invasion in A549 cells induced by Thapsigargin (**A**) Immunoblotting of PIMT and E-cadherin in A549 cells treated with 0.1 µM of Tg, and PIMT expression and empty vectors. (**B, C**) Intensity of PIMT and E-cadherin in A549 cells treated with Tg, and PIMT expression and empty vectors. ^*^indicates *p* < 0.05, ^¶^ indicates *p* < 0.05.

Moreover, we explored whether overexpression of PIMT relieves the decrease in E-cadherin expression of A549 cells induced by Tg stress (Figure 5A–5C). Transfection using a PIMT vector caused a significant increase in PIMT expression compared to that in the control transfected with an empty vector (Figure 5A and 5B). Overexpression of PIMT recovered the protein level of E-cadherin that was decreased by 0.1 µM of Tg stress and increased E-cadherin levels to near baseline without Tg stress (Figure 5A and 5C).

### Supplemental expression of PIMT reduces EMT and cell invasion in A549 cells induced by hypoxia

**Figure 6:**
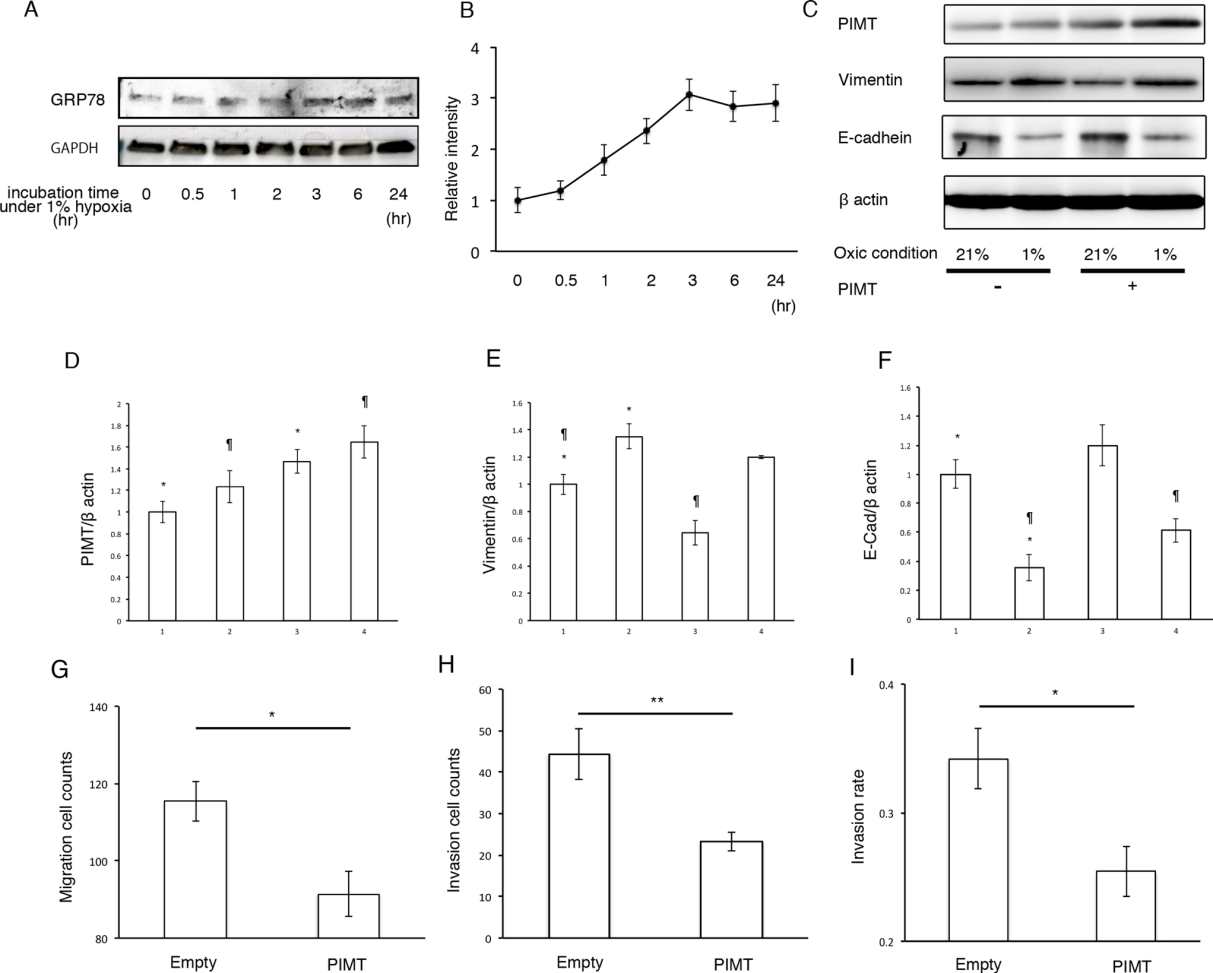
Supplemental expression of PIMT reduces EMT and cancer invasion in A549 cells induced by hypoxic conditions (**A, B**) Immunoblotting and relative intensity of GRP78 in A549 cells under normal hypoxic (1% O_2_) conditions. (**C**) Immunoblotting of PIMT, vimentin, and E-cadherin in A549 cells under normal (21% O_2_) and hypoxic (1% O_2_) conditions treated with and without PIMT vector. (**D–F**) Intensity of PIMT, vimentin, and E-cadherin in A549 cells treated with and without PIMT vector. Line 1: normoxia + empty vector, Line 2: hypoxia + empty vector, Line 3: normoxia + PIMT vector, Line 4: hypoxia + PIMT vector. (**G**) Migration cell count, (**H**) Invasion cell count, and (**I**) Invasion rate in A549 cells under hypoxic conditions with empty and PIMT vector. ^*^indicates *p* < 0.05, ^**^indicates *p* < 0.01 and ^¶^ indicates *p* < 0.05.

Next, we explored whether hypoxic conditions can induce EMT in A549 and H441 cells. Under hypoxic conditions (1% O_2_ concentration), the expression of GRP78 in A549 but not in H441 cells showed a significant increase in a time-dependent manner, indicating that hypoxic conditions induce ER stress in A549 cells (Figure 6A and 6B, and Supplementary Figure 3). Furthermore, we tested whether overexpression of PIMT reduces EMT in A549. Overexpression of PIMT under normoxic conditions induced a decrease in vimentin expression with an increase in E-cadherin levels in A549 cells (Figure 6C–6F). Similarly, overexpression of PIMT induced a decrease in vimentin expression with an increase in E-cadherin levels in A549 cells induced by 1% O_2_ conditions.

Moreover, we explored whether PIMT expression is involved in the mobility of A549 cells under hypoxic conditions (Figure 6G–6I). Migration and invasion quantities under hypoxic conditions were significantly decreased by overexpression of PIMT compared to those in the negative control. In addition, invasion rates were also decreased by overexpression of PIMT.

### HIF1α regulates EMT and cell invasion in A549 cells induced by deficiency of PIMT expression under ER stress

**Figure 7:**
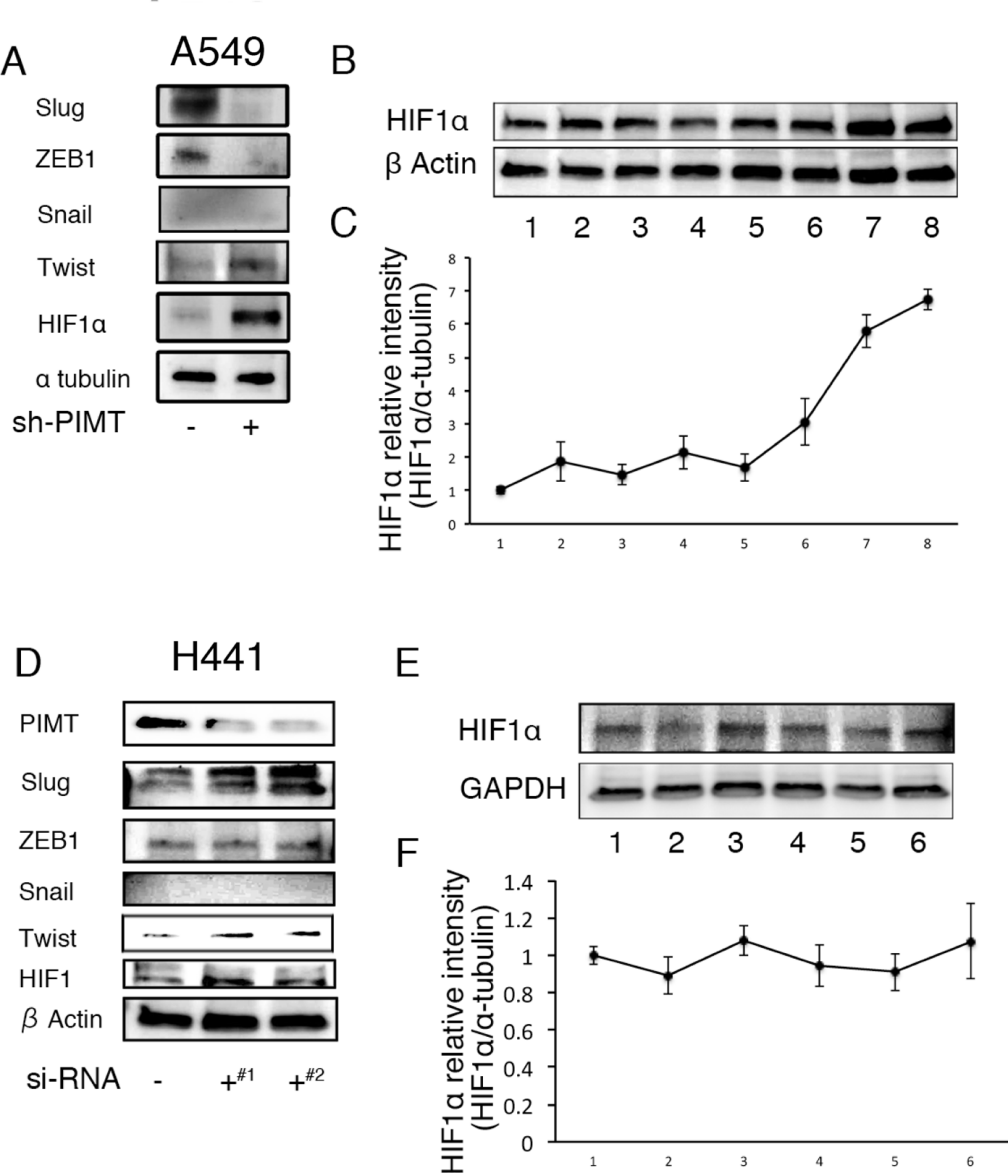
Increased expression of HIFα and/or Twist in A549 and H441 cells induced by the inhibition of PIMT and Thapsigargin (**A**) Immunoblotting of Slug, ZEB1, Snail1, Twist, and HIF1α in A549 sh-PIMT and sh-control cells. (**B, C**) Immunoblotting and relative intensity of HIF1α in A549 cells treated with Tg. (**D**) Immunoblotting of Slug, ZEB1, Snail1, Twist, and HIF1α in si-control cells and si-PIMT H441 cells. (**E, F**) Immunoblotting and relative intensity of HIF1α in H441 cells treated with Tg. #1 and #2 indicates si-RNA of J-010000-05-0002 and J-010000-07-0002, respectively.

**Figure 8:**
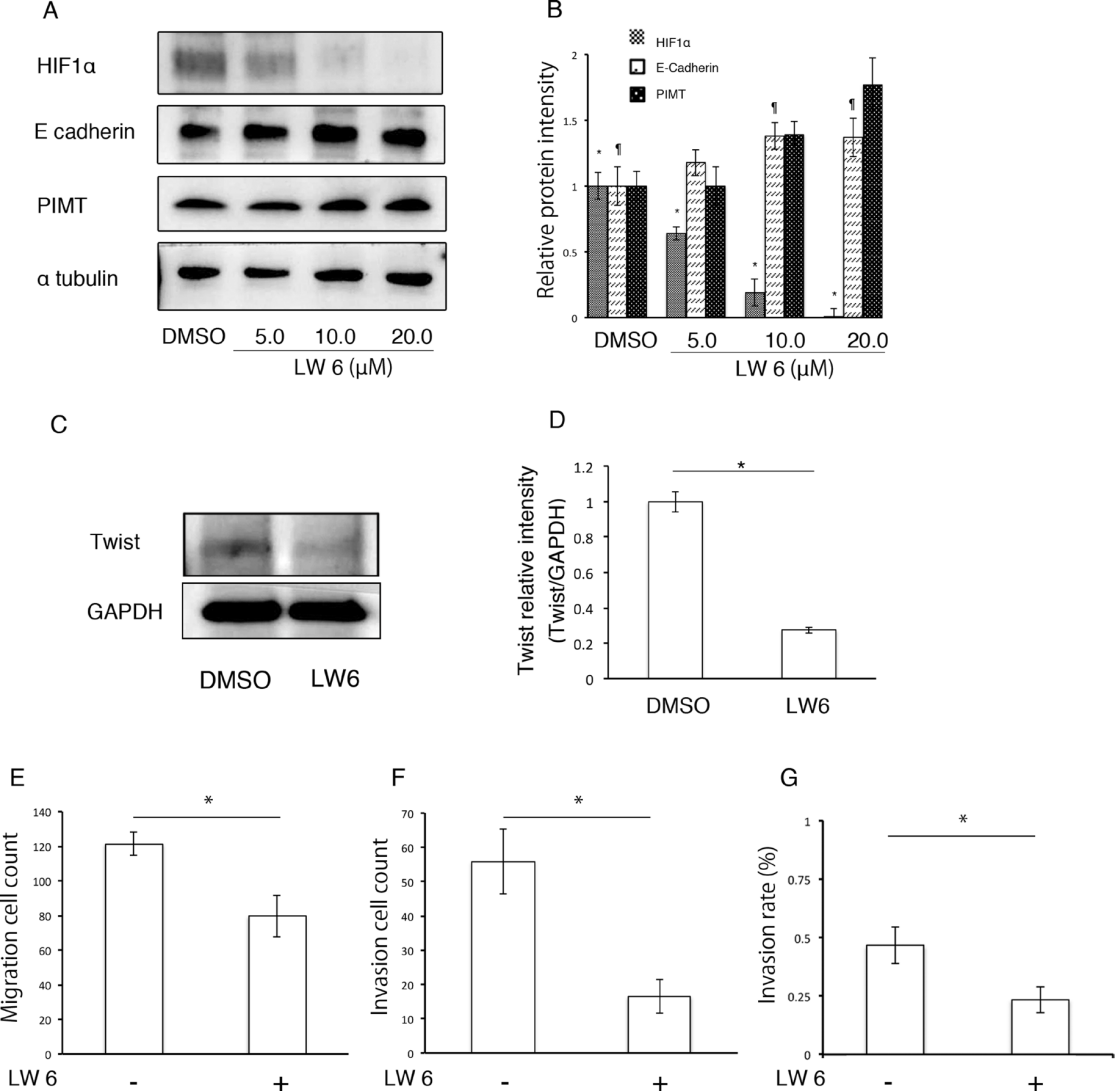
HIF1α inhibitor decreases EMT and cancer invasion in A549 cells induced by inhibition of PIMT expression (**A** and **B**) Immunoblotting and intensity of HIF1α, E-cadherin, and PIMT in A549 cells treated with LW6 under 0.1 µM of Tg. **(C** and **D**) Immunoblotting of Twist in sh-PIMT A549 cells treated with LW6. (**E**) Migration cell count, (**F**) Invasion cell count, and (**G**) Invasion rate in A549 sh-PIMT cells treated with LW6. ^*^indicates *p* < 0.05 and ^¶^ indicates *p* < 0.05.

We evaluated which transcription factors participate in EMT and cell invasion in A549 cells induced by ER stress. Compared with sh-control and si-control cells, protein levels in HIF1α and Twist were higher in sh-PIMT A549 and si-PIMT H441 cells (Figure 7). No difference was detected in HIF1α expression of the other four cell lines between si-control and si-PIMT cells (Supplementary Figure 4). Slug expression in si-PIMT H441 cells, but not in sh-PIMT A549 cells, was increased relative to control cells, while Snail was expressed in neither A549 nor H441 cells. Zeb-1 was somewhat decreased in sh-PIMT A549 and si-PIMT H441 cells. The relationship between Tg stress and HIF1α expression in A549 and H441 cells was then explored. Protein levels of HIF1α increased gradually with increasing concentrations of Tg in A549 cells, while H441 showed minimal changes in HIF1α levels (Figure 7). Further, we explored whether LW6, an HIF1α inhibitor, inhibits EMT of A549 cells induced by Tg stress. LW6 considerably suppressed HIF1α expression in A549 cells treated with 0.1 µM of Tg in a dose-dependent manner, while protein levels of E-cadherin were gradually increased (Figure 8A and 8B). In addition, LW6 decreased the protein levels of Twist (Figure 8C and 8D). These results indicate that HIF1α signals are involved in EMT of A549 cells induced by Tg stress. Moreover, we clarified that inhibition of HIF1α reduces cell mobility in sh-PIMT A549 cells. Compared to the DMSO control, LW6 administration significantly reduced the migration and invasion capabilities of sh-PIMT A549 cells (Figure 8E–8G).

## DISCUSSION

In the present study, we demonstrated that EMT and increased cell mobility of A549 cells were induced by inhibition of PIMT using si-RNA and sh-RNA, as well as Tg stress and hypoxic conditions. Tg stress and hypoxia increased the GRP78 levels of A549 cells in a dose- and time-dependent manner, respectively. The decreased E-cadherin levels induced by Tg and hypoxia were relieved by overexpression of PIMT. These results indicate that PIMT contributes to the conservation of epithelial properties in A549 cells against Tg- and hypoxic stresses. It has been reported that several mediators and/or conditions induce EMT in A549 cells [15–18]. Particularly, TGFβ is a powerful inducer of EMT in A549 cells [14]. In the present study, however, inhibited expression of PIMT was related to a decrease in TGFβ, indicating that EMT related to PIMT inhibition occurs independently of TGFβ.

The remaining cell lines did not show similar responses to the inhibition of PIMT by si-RNA, Tg, and hypoxic stresses. The inhibition of PIMT expression by si-RNA also facilitated EMT and cell mobility in H441 similarly to in A549 cells, and GRP78 levels were increased in H441 cells by Tg stress. In H1650 cells, the decreased levels of E-cadherin expression were detectable by the inhibition of PIMT using si-RNA, although neither vimentin expression nor cell mobility was changed. However, H441 and H1650 cells did not show a decrease in E-cadherin expression by Tg and/or hypoxia. Our results indicate that PIMT is involved in preserving the epithelial properties of H441 and H1650 cells, but we could not determine other ER stressors that induce EMT in these cell lines. In the remaining three cell lines, E-cadherin expression was not decreased by the inhibition of PIMT using si-RNA. PIMT was not involved in the conservation of epithelial properties in the three cell lines.

We previously found that higher PIMT expression in cancer cells in immunohistochemistry is correlated with poor prognoses in patients with surgically resected lung adenocarcinoma. In the present study, we demonstrated that deficiency of PIMT supplemental expression induced EMT in A549 cells. These findings appear to be inconsistent in terms of the prognostic values of PIMT, as EMT was shown to be related to lymph node and distant metastasis [19]. We formed a rational hypothesis to explain these findings; the cancer cells that establish a compensatory mechanism of PIMT expression against increased ER stresses may remain at primary lesions with conserved epithelial properties and continuously repeat self-proliferation. In contrast, cancer cells that fail to establish a compensatory mechanism of PIMT expression may enter EMT to escape from stresses, followed by invasion into the cancer stroma. Stronger PIMT expression would be beneficial in A549, H441, and H1650 cells, in terms of protection from EMT and cancer invasion, although higher PIMT expression in the other cell lines may be harmful in terms of induction of cancer cell apoptosis and anti-cancer proliferation. Increasing evidence supports that multiple cell lines derived from the same histopathological type of cancer can behave differently [20]. In this context, our results indicate the necessity of multiple treatment strategies in lung adenocarcinoma.

Some transcription factors including Slug, Zeb-1, Twist, Snail, and HIF1α, were predicted to be associated with EMT in lung adenocarcinoma based on both immunohistochemistry and *in vitro* experiments [21–25]. In the present study, increased expression of HIF1α and Twist was associated with EMT in A549 and H441 cells induced by PIMT inhibition. In addition, Tg stress increased HIF1α expression levels in A549 cells in a dose-dependent manner. Moreover, the HIF1α inhibitor reduced EMT, cancer invasion, and the protein levels of Twist in sh-PIMT A549 cells. These findings indicate that HIF1α is at least partially involved in PIMT-related EMT in A549 cells via Twist signals. Recent reports provide mechanistic evidence of an association between HIF1α-Twist signals and EMT, thus supporting our findings [26–31]. In particular, some papers demonstrated that HIF1α directly binds to the proximal promoter site of Twist in chromatin immunoprecipitation assay, and then regulates Twist expression [26, 32]. Chemotherapy agents are generally used to induce the apoptosis of cancer cells as an anti-cancer therapy. It has been reported that chemotherapy causes ER stress in cancer cells [33]. Thus, cancer chemotherapy may induce EMT of cancer cells. Therefore, inhibiting HIF1α in combination with chemotherapy may offer a novel treatment target for lung adenocarcinoma to prevent both cancer proliferation and EMT. Further studies are required to determine the mechanism by which PIMT regulates HIFα expression.

Previous studies reported that wild-type p53 generally suppresses EMT in cancer cells, and EMT in cancer cells is caused by the inhibition of wild-type p53 or induction of mutant p53 [34, 35]. The expression of mutant p53 competes with that of wild-type p53 [36]. Lee *et al.* reported that the inhibitory expression of PIMT using siRNA induced an increase in the expression of wild-type p53 [11]. We found that the intensity of wild-type p53 expression was not clearly correlated with Tg dose. Therefore, involvement of p53 in the mechanism of EMT in A549 cells appeared to be limited, although we could not precisely determine how p53 participated in the EMT.

Ryu *et al.* previously reported that PIMT showed a significantly increasing correlation with the phosphorylation of ERK1/2 during incubation of MDA-MB-231 cells, a breast cancer cell line. Under detached conditions, but not attached conditions, PIMT siRNA blocked the phosphorylation of ERK and expression of EMT proteins, which is inconsistent with the results of the present study [12]. In our study, EMT induced by the inhibition of PIMT was confirmed in multiple cell lines of lung adenocarcinoma. It remains unclear whether the discrepancy is attributable to the difference in cancer types or cell conditions.

We found that the deficiency of compensatory PIMT expression under increased ER stress induces EMT and cancer invasion in lung adenocarcinoma via a HIF1α signal. Our results provide insights into the pathogenic perspective of disease progression and indicate the necessity of multiple treatment strategies in lung adenocarcinoma. Inhibition of PIMT and HIF1α may be a novel treatment target for lung adenocarcinoma.

## MATERIALS AND METHODS

### Cell lines

All cell lines (A549, H441, H460, H1650, Calu 1, and Calu 6) were obtained from the ATCC (Manassas, VA, USA). A549 and H441 cells represented preinvasive stages, while H460, H1650, Calu 1, and Calu 6 cells represented advanced types [37–40]. Cells were grown in RPMI1640 media supplemented with 10% (v/v) fetal bovine serum (FBS), penicillin, and streptomycin, in a humidified atmosphere of 5% CO_2_ and 95% air at 37° C. The cells were tested and authenticated by short tandem repeat analysis. Stably transformed cells were selected using geneticin (1000 μg/mL) and collected by cell sorting three times (>95% purified) with a fluorescent protein marker (ZsGreen1) to improve cell purity [41].

### Antibodies and reagents

Anti-PIMT (Abcam PLC, Cambridge, UK, 97446, 1:2000 dilution), anti-E-cadherin (Santa Cruz Biotechnology Inc., Santa Cruz, USA, sc-7870, 1:200 dilution), anti-vimentin (sc-7557, 1:250 dilution), anti-α tubulin (sc-5546, 1:400 dilution), anti-GRP78 (sc-1051, 1:200 dilution), anti-p53 (sc-1616, 1:200 dilution), anti-β actin (sc-32293, 1:200 dilution), anti-GAPDH (sc-25778), anti-Zeb1 (Atlas Antibodies AB, Stockholm, Sweden, HPA027524, 1:500 dilution), anti-Slug (sc-15391, 1:200 dilution), anti-Twist (Proteintech, Rosemont, IL, USA, 1:500 dilution), anti-Snail1 (Proteintech, 1:1000 dilution), anti-HIF1α (Novus Biologicals, Minneapolis, USA, NB100-479, 1:1000 dilution), and anti-HIF1α (Abcam, ab51608, 1:1000 dilution) antibodies were used in this study. Particularly, we used E-cadherin and vimentin to detect epithelial and mesenchymal properties in the cell lines. E-cadherin is a calcium-dependent transmembrane glycoprotein that mediates cell-cell adhesion in the polarized epithelium [42]. The loss of its expression is a hallmark of EMT [43]. Vimentin is a major marker of mesenchymal properties. Thapsigargin (Tg) was purchased from Wako Pure Chemical Industries, Ltd. (Osaka, Japan). Tunicamycin (Tn) was purchased from Sigma-Aldrich (St. Louis, MO, USA). LW6, an inhibitor against HIF1α, was purchased from Merck Millipore (Darmstadt, Germany).

### Knockdown and overexpression of PIMT

Knockdown of PIMT in A549, H441, H460, H1650, Calu 1, and Calu 6 cells and overexpression in A549 cells were carried out in a 6-well format and the cells were transfected according to standard methods. Briefly, cells were seeded in RPMI1640 media containing 10% FBS with/without antibiotics. After 24 h, the cells were transfected with selected siRNA fragments or vectors in Opti-MEM media (Thermo Fisher Scientific, Waltham, MA, USA) using Lipofectamine RNAiMAX or Lipofectamine 3000 (Invitrogen, Carlsbad, CA, USA). Stealth RNAi siRNA for PIMT (J-010000-05-0002, -06-, -07, -08-; GE Healthcare, Little Chalfont, UK) were transfected with Lipofectamine RNAiMAX according to the manufacturer’s instructions. Stealth RNAi siRNA Negative Control Low GC Duplex No.3 (GE Healthcare) was used as a scrambled control throughout the experiment. PIMT knockdown and overexpression were confirmed by western blotting analysis. Each experiment was performed at least in triplicate three times.

### Construction of PIMT-Cont shRNA and PIMT-KD shRNA vectors

We employed a Pol III terminator sequence (5′-TTTTTT-3′) in the hairpin loop region as a control for gene silencing experiments to select cells with stable expression. This sequence has previously been used as a negative control to generate brain-specific MAPK-silencing transgenic mice [44]. A pENTR™/H1/TO vector (Thermo Fisher Scientific) was used to generate PIMT-Cont shRNA and PIMT-KD shRNA. To construct PIMT-Cont, two oligonucleotide sequences, 5′-GTA TGA CAA GCT ACA AGA TTTTTT CTT GTA GCT TGT CAT ACT GC-3′ (underlined sequence: Pol III terminator) and 5′-GCA GTA TGA CAA GCT ACA AG AAAAAA TCT TGT AGC TTG TCA TAC TGC-3′, were synthesized and annealed for ligation into the pENTR™/H1/TO vector according to the manufacturer’s instructions. The PCMT1-Cont vector contained a Pol III terminator sequence in place of the hairpin loop and was designed to prevent expression of the complete PIMT-shRNA. To construct PIMT-KD, two oligonucleotide sequences, 5′-GCA GTA TGA CAA GCT ACA AGA CGAA CTT GTA GCT TGT CAT ACT GC-3′ (double underlined sequence: hairpin loop) and 5′-GCA GTA TGA CAA GCT ACA AG TTCG TCT TGT AGC TTG TCA TAC TGC-3, were synthesized and annealed for ligation into pENTR™/H1/TO according to the manufacturer’s instructions.

### Construction of PIMT vectors

A PIMT mRNA expression vector was generated by alternative splicing [41], and a pIRES2-ZsGreen1 vector was used to express PIMT. PCMT1-ER cDNA was amplified by PCR using the forward primer 5′-CG GAA TTC ATG CCG GGA GCG CGC AGT GGC GGC AGC-3′ (underlined sequence is an EcoRI cleavage site) and reverse primer 5′-GC GGA TCC TTA CAA TTC ATC CCT GGA CCA CTG C-3′ (underlined sequence is a BamHI cleavage site). The amplified PCR products were incubated at 37° C with EcoRI and BamHI, after which they were purified using a Qiagen gel extraction kit (Hilden, Germany). The purified and excised products were ligated into the EcoRI/BamHI sites of the pIRES2-ZsGreen1 expression vector.

### Immunoblotting

Protein aliquots of 15 µg each were resolved by SDS polyacrylamide gel (Bio-Rad Laboratories, Hercules, CA, USA) electrophoresis and transferred to polyvinylidene difluoride membranes (Bio-Rad). After washing three times, membranes were incubated with Blocking One (Nacalai Tesque, Inc., Kyoto, Japan) for 1 h at room temperature, and incubated overnight at 4° C with primary antibodies. Thereafter, membranes were washed three times and incubated for 1 h at room temperature with secondary Ab [horseradish peroxidase- conjugated species-specific Ab]. Immunoreactive bands were visualized with Clarity Western ECL Substrate (Bio-Rad). Each experiment was performed at least three times independently.

### Cell morphology analysis

A549 cells with PIMT shRNA or control shRNA were stained with Cytopainter Phalloidin-iFluor 555 conjugate (Abcam, 97446) and DAPI (Dojindo, Tokyo, Japan) to visualize the actin filaments and nuclei.

### Cell migration and invasion assay

Cell migration and invasion were examined by Matrigel (Promega, Fitchburg, WI, USA) invasion assays according to the manufacturer’s instructions. Briefly, wild-type A549, H441, and H1650 cells or sh-PIMT and sh-control stable A549 cells at a density of 2.5 × 10^4^ per well were placed in the upper BD Biocoat Matrigel Invasion Chamber (insert 6.4-mm diameter, 8-mm pore size; BD Bioscience, Franklin Lakes, NJ, USA) in RPMI1640 media without serum. Background migration towards media with 10% FBS was subtracted. The number of transmigrated cells was counted after 24 h. Each experiment was repeated at least three times independently.

### Cell viability assay

A549 cells with PIMT shRNA and control cells were reseeded at 5 × 10^3^ per well in 96-well plates, and incubated in antibiotic-containing RPMI1640 with 10% FBS. After 24 h of incubation, cisplatin (8.0 µg/mL), a key drug in chemotherapy for non-small cell lung carcinoma, was added to each well and incubation was continued for an additional 24 and 48 h. These cells were then used for the proliferation assay, which was measured using Cell Counting Kit-8 (Dojindo). An aliquot of WST-8/1-Methoxy PMS solution was added to each well followed by incubation for 2 h at 37° C according to the protocol. Absorbance was measured with a Multiskan FC microplate reader (Thermo Fisher Scientific) at test and reference wavelengths of 450 nm. The percentage of growth is shown relative to untreated controls. Each sample was assayed in duplicate, with each experiment repeated at least three times independently.

### Toxicity assay

To assess whether cell transformation was effectively induced by ER stress initiated by Tg or Tn, A549, H441, H1650, and H460 cells were seeded into 6-well dishes (Iwaki, Chiba, Japan) at 5.0 × 10^5^ cells/dish and treated with increasing concentrations of 1.0 × 10^-4^–1.0 μM or 1.0×10^-4^–1.0 μg/mL for 16 h, respectively. To assess whether LW6 could inhibit cell transformation, A549 and H441 cells were seeded into 6-well dishes at 1.0 × 10^6^ cells/dish with 2.0 mL medium containing LW6 for 16 h.

### Statistical analysis

Statistical significance was evaluated using Mann-Whitney *U* test. Statistical analyses were based on SPSS software (SPSS, Inc., Chicago, IL, USA).

## SUPPLEMENTARY MATERIALS FIGURES


