# Supplementary material for: Deficiency of protein-L-isoaspartate (D-aspartate) O-methyl-transferase expression under endoplasmic reticulum stress promotes epithelial mesenchymal transition in lung adenocarcinoma
Source: Oncotarget. 2018 Jan 27;9(17):13287–300. doi: 10.18632/oncotarget.24324 (PMC5862578; doi:10.18632/oncotarget.24324)
Supplement: Supplementary file 1 [file oncotarget-09-13287-s001.pdf]

Deficiency of protein-L-isoaspartate (D-aspartate) *O*-methyltransferase expression under endoplasmic reticulum stress promotes epithelial mesenchymal transition in lung adenocarcinoma

SUPPLEMENTARY MATERIALS

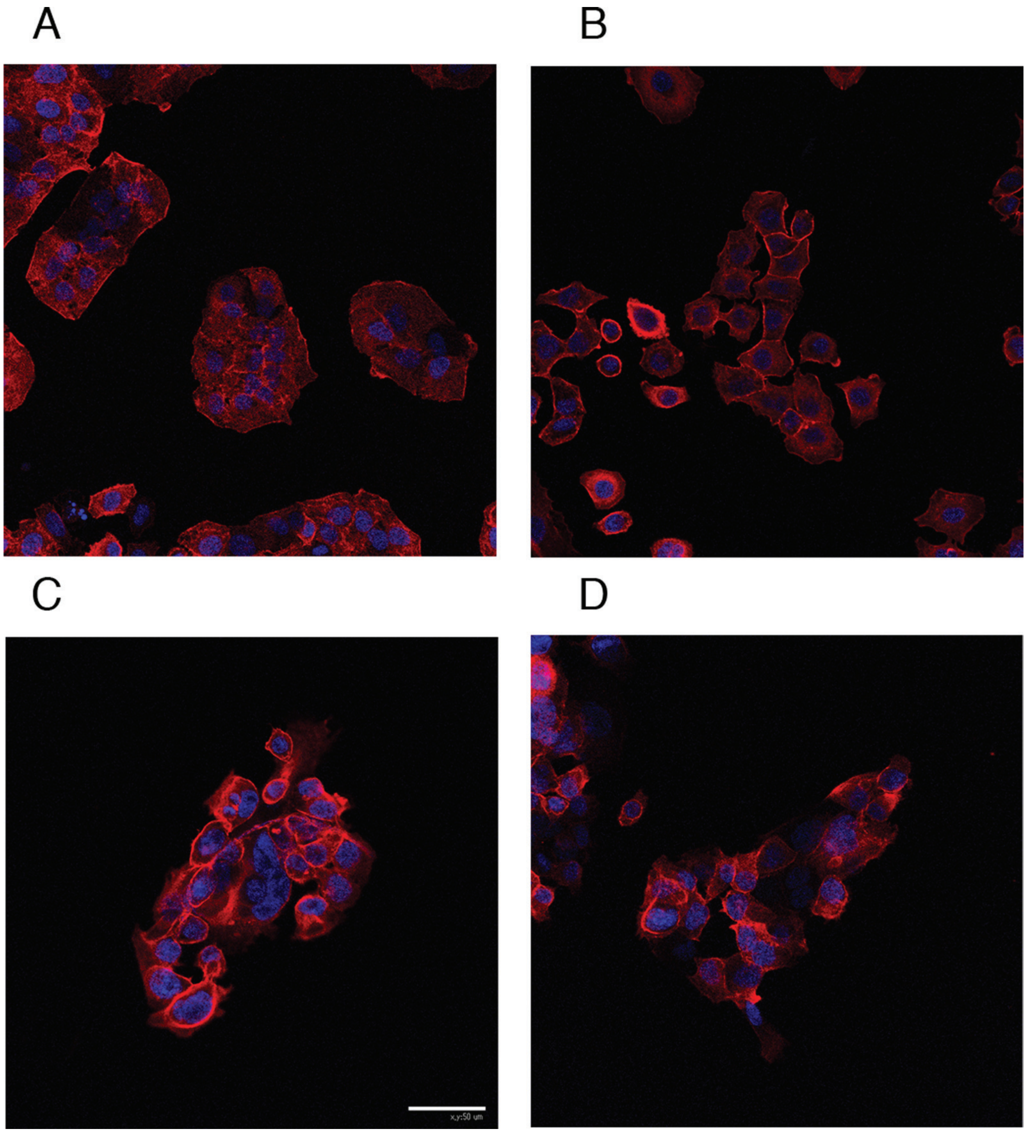

Supplementary Figure 1: Morphologic changes in A549 and H441 cells by inhibition of PIMT using si-RNA. (**A, B**) Si-control and si PIMT A549 cells. (**C, D**) Si-control and si PIMT H441 cells. Scla bar, 50 µm.

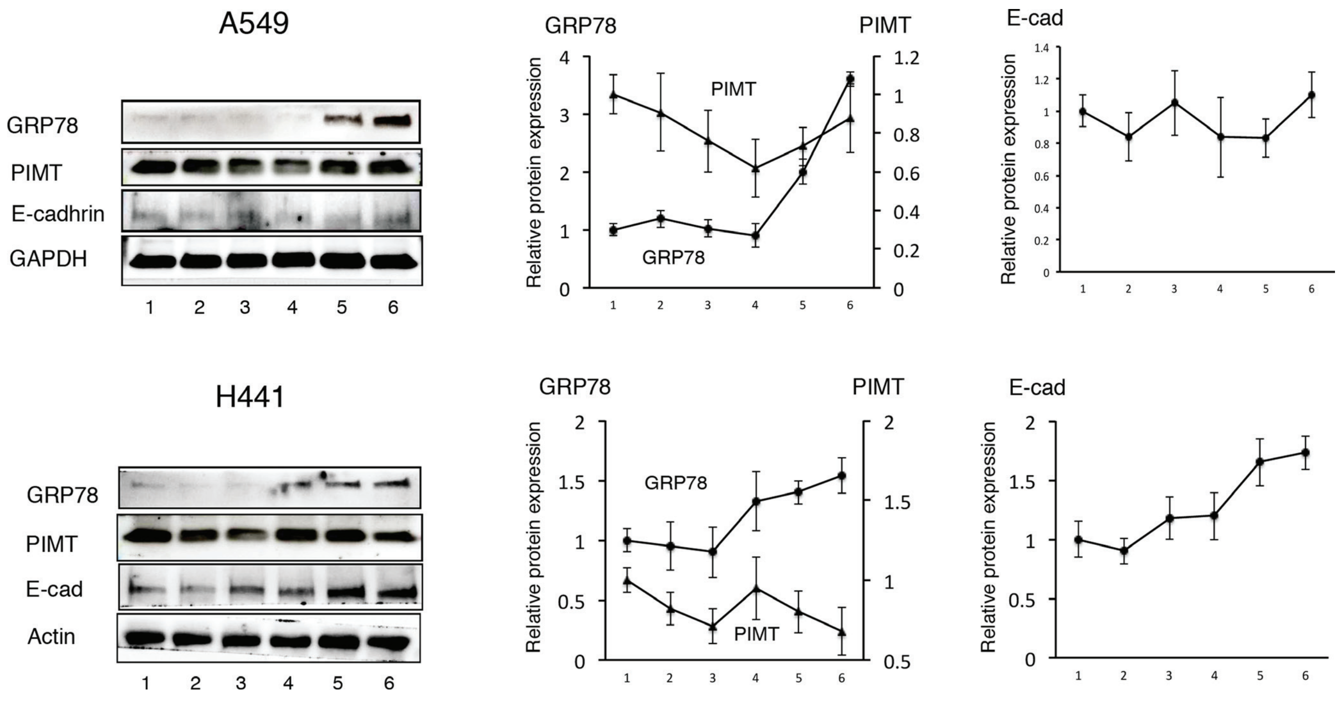

Supplementary Figure 2: Response of lung adenocarcinoma cell lines to tunicamycin. (**A**) Immunoblotting of GRP78, PIMT, and E-cadherin in A549 cells treated with tunicamycin (Tn). Line 1, DMSO; Line 2, 1.0×10^{-4} µg/mL; Line 3, 1.0×10^{-3} µg/mL; Line 4, 1.0×10^{-2} µg/mL; Line 5, 0.1 µg/mL; Line 6, 1.0 µg/mL Line 7 of Tn. (**B**) Intensity of GRP78 and PIMT in A549 cells treated with Tn. (**C**) Intensity of E-cadherin in A549 cells treated with Tn. (**D**) Immunoblotting of GRP78, PIMT, and E-cadherin in H441 cells treated with Tunicamycin (Tn). Line 1, DMSO; Line 2, 1.0×10^{-4} µg/mL; Line 3, 1.0×10^{-3} µg/mL; Line 4, 1.0×10^{-2} µg/mL; Line 5, 0.1 µg/mL; Line 6, 1.0 µg/mL Line 7 of Tn. (**E**) Intensity of GRP78 and PIMT in H441 cells treated with Tn. (**F**) Intensity of E-cadherin in H441 cells treated with Tn.

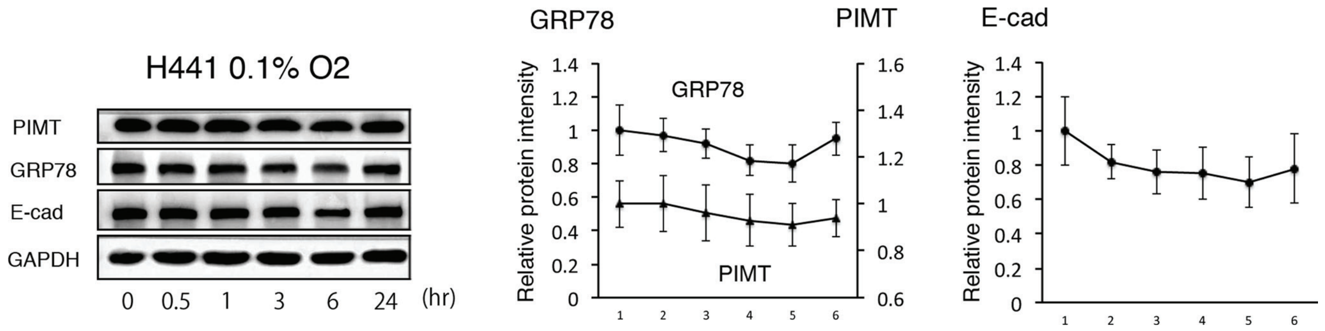

Supplementary Figure 3: GRP78 is not increased in H441 cells under hypoxic condition. (**A**) Immunoblotting of GRP78, PIMT and E-cadherin in H441 cells under normal hypoxic (1% O_2) conditions. (**B**) Relative intensity of GRP78 and PIMT in H441 cells under normal hypoxic (1% O_2) conditions. (**C**) Relative intensity of E-cadherin in H441 cells under normal hypoxic (1% O_2) conditions.

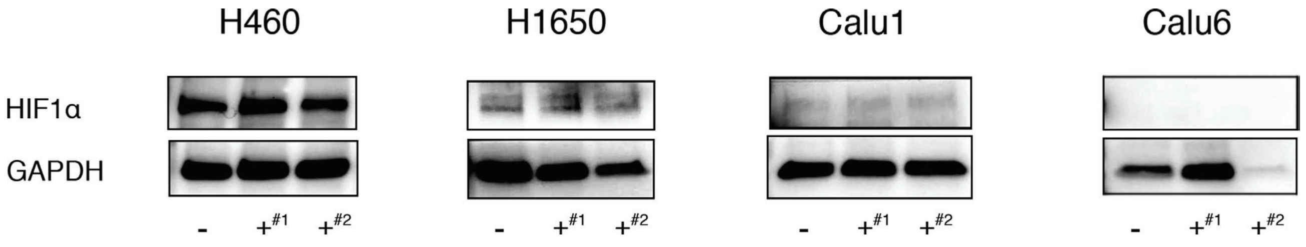

Supplementary Figure 4: HIF1α level is not increased in H1650, H460, Calu1, and Calu 6 cells in response to PIMT inhibition using si-RNA. Immunoblots of HIF1α in the four cell lines. PIMT si-RNA anti-sense; [¶]J-010000-05-0002 and [§]J-010000-07-0002.
